# Enterprise retail price prediction method based on improved HPO-LSTM algorithm

**DOI:** 10.1371/journal.pone.0339155

**Published:** 2026-01-02

**Authors:** Wei Zhu

**Affiliations:** Jining College, Qufu, China; Ariel University, UNITED KINGDOM OF GREAT BRITAIN AND NORTHERN IRELAND

## Abstract

In modern market economy, accurate price prediction plays a pivotal role in inventory management, marketing strategy formulation, and profit maximization for enterprises. To optimize resource allocation and improve economic efficiency, this research proposes an optimization algorithm that combines Hunter-Prey optimization algorithm and long short-term memory network, while introducing attention mechanism and Q-learning algorithm to optimize the model. In the research results, the improved Hunter-Prey optimization-long short-term memory algorithm showed significantly better prediction accuracy than the control model, with a root mean square error of 0.48 and a mean absolute error of 0.20. At the same time, after the algorithm converged, the accuracy tended to 0.972 and the recall tended to 0.921. In practical business applications, this algorithm could significantly reduce inventory costs (15.2%) and promotion costs (20.3%), while increasing sales revenue (15.4%) and profits (20.4%). The results indicate that the improved Hunter-Prey optimization-long short-term memory algorithm can effectively reduce prediction errors and strengthen the robustness and adaptability of the model. Research can provide enterprises with an efficient and accurate retail price prediction tool, which can help optimize inventory management, reduce resource waste, and enhance market competitiveness.

## 1 Introduction

In the rapidly developing market economy, the changes in retail prices of enterprises are influenced by massive complex factors, including product costs, consumer demand, economic environment, and promotional strategies. Accurately predicting retail prices not only helps companies optimize inventory management and develop reasonable pricing strategies, but also enhances market competitiveness and economic benefits [1,2]. As the big data and artificial intelligence technology develop, more and more research is focusing on using advanced computer algorithms and models to raise the accuracy and efficiency of price forecasting. However, traditional prediction methods often struggle to cope with complex market environments and dynamic price fluctuations, especially in situations where multiple factors interact [3,4]. The so-called complex market environment is mainly reflected in the following aspects. Firstly, the acceleration of globalization has made market competition increasingly fierce. Enterprises not only have to contend with competition from domestic peers but also face competitive pressure from the international market, which has led to a significant increase in market demand uncertainty. Secondly, fluctuations in the economic situation, such as changes in economic cycles, inflation or deflation, etc., can have a significant impact on consumers’ purchasing power and willingness, and thereby affect the retail prices of goods. Dynamic price fluctuations mainly come from two aspects. Firstly, fluctuations in raw material prices will directly affect the cost of goods, thereby leading to changes in retail prices. For instance, the increase in oil prices will lead to a rise in the cost of chemical products related to oil, which in turn will drive up their retail prices. Secondly, seasonal changes in consumer demand can also cause price fluctuations. Take the clothing industry as an example, spring and autumn are peak sales seasons, with strong demand and relatively high prices. Winter and summer, on the other hand, are the off-seasons for sales, with reduced demand and corresponding price drops. In addition, sudden events, such as natural disasters and public health incidents, can also cause short-term and sharp fluctuations in prices.

Against the backdrop of the current complex market environment and dynamic price fluctuations, traditional prediction methods often struggle to cope. For instance, classic statistical methods, such as the ARIMA model in time series analysis, perform well when dealing with stationary time series data, but their prediction accuracy drops significantly when dealing with nonlinear and non-stationary time series data. While traditional machine learning methods, such as linear regression and decision trees, can handle a certain degree of nonlinear relationships, their performance is also poor when dealing with complex multi-factor interactions and long-term dependencies of time series data. Therefore, to enhance the accuracy and efficiency of price prediction and provide enterprises with a more scientific and precise decision-making basis, it is particularly necessary to research and develop better prediction algorithms. Long Short-Term Memory (LSTM) networks, as an effective recursive neural network structure, are widely used in price prediction. This model can capture long-term dependencies in time series data through its unique gating mechanism, but the selection of hyperparameters and optimization problems during model training are still the focus of research. In addition, although LSTM performs well in processing time series data, it may still suffer from insufficient prediction accuracy due to data noise and model complexity issues when facing complex market environments [5,6].

Currently, scholars have conducted research on price prediction. Kożuch A et al. proposed a comprehensive prediction model combining artificial neural networks and time series for existing wood price prediction methods, which consists of two artificial neural network structures: multilayer perceptron and radial basis function. Compared with classical time series models, the results showed that this model had stronger adaptability in different types of wood and price fluctuations, and performed well in predicting price extremes [7]. Wang K et al. proposed a quantitative model for electricity price prediction grounded on random forest and improved Mahalanobis distance to address the difficulty of electricity price prediction due to the widespread application of renewable energy networks. The model used variational mode decomposition (VMD) technology to divide electricity price sequence data into intrinsic mode functions and residuals of different frequencies, to minimize data noise and volatility. The outcomes denoted that the hybrid model could significantly improve the predictive ability of electricity prices while reducing prediction errors, and its performance was significantly better than other control models [8]. Xu X et al. raised a neural network model for predicting office property price indices in response to the quick advancement of the Chinese housing market over the past decade. This model explored the setting of different algorithms, delays, hidden neurons, and data segmentation ratios to achieve pure technical prediction of the Chinese office real estate market. The results indicated that the mean relative RMSE of the model for predicting housing prices was only 1.45% [9]. Lahaghi S A M et al. proposed a distributed node marginal price evaluation model based on the risk-sensitive cheetah hunter optimization algorithm in view of the current situation of distributed generation pricing optimization in smart distribution networks. This model combined the scenario analysis method of the information gap decision-making theory, simulated the uncertainty of the electricity market price through two scenarios of risk aversion and risk tolerance, and utilized the global and local search capabilities of the Cheetah Hunter algorithm to optimize the marginal price of distributed nodes. The results showed that this method could effectively reduce network losses under different market conditions and provide higher profits for distributed generation units at the same time [10]. Finally, a simple model based on four delays and two hidden neurons was constructed. The results showed that the relative root mean square error of the model in the training, verification and testing stages was all lower than 1.80%, and the average absolute percentage error was lower than 1.30%. Furthermore, this model passed the modified Diebold-Mariano test at the 1% significance level, demonstrating better predictive performance than the linear autoregressive model [11]. Yue W et al. proposed a Transformer-based hybrid model in response to the current situation of carbon price prediction. This model first used VMD to decompose the original carbon price sequence into multiple sub-sequences, then used the Transformer model to predict each sub-sequence, and finally reconstructed the prediction results to obtain the final predicted carbon price value. The results showed that the multi-step prediction and interval prediction performance of this model on two real carbon market datasets were superior to those of traditional models such as ARIMA, SVR, and LSTM, which proves the effectiveness and superiority of the Transformer model in the field of carbon price prediction [12]. Sefer et al. proposed a temporal deep learning model integrating Graph Neural Networks (GNNS) in response to the current situation of financial asset price prediction. The results showed that this model was significantly superior to the traditional sequence model in capturing the complex correlations and dynamic evolution among assets, and improved the prediction accuracy of prices and their directions of rise and fall [13].

Although existing research has realized certain results in price prediction, there are still limitations in complex market environments, multi-factor interactions, and model optimization. At the same time, due to the complexity of multi-source data such as product prices, promotional activities, and economic indicators, coupled with the dynamic and nonlinear characteristics of time series data, traditional linear models are difficult to accurately capture price fluctuations. Based on this, a Hunter-Prey Optimization Long Short-Term Memory (HPO-LSTM) network is proposed for predicting retail prices in enterprises. The innovation of the research lies in the combination of the HPO algorithm, the attention mechanism, and Q-learning, forming a new retail price prediction model. The HPO algorithm is used to optimize the hyperparameters of LSTM, improving the prediction accuracy of the model. The attention mechanism enhances the model’s ability to capture key information in time series. The Q-learning algorithm optimizes the training process of the model by dynamically adjusting the learning rate and hyperparameters. In the task of time series prediction, the application value of the attention mechanism lies in its ability to capture the key time steps in the time series, thereby enhancing the model’s modeling ability for long-term dependencies. Meanwhile, the Q-learning algorithm can be used to dynamically adjust the hyperparameters of the model, such as the learning rate, to optimize the training process of the model. The research targets to improve the accuracy and robustness of retail price prediction for enterprises through an improved HPO-LSTM algorithm, to provide scientific basis for relevant decision-making for enterprises.

## 2 Methods and materials

### 2.1 Factors affecting sales in the retail market of enterprises and data processing

**Fig 1:**
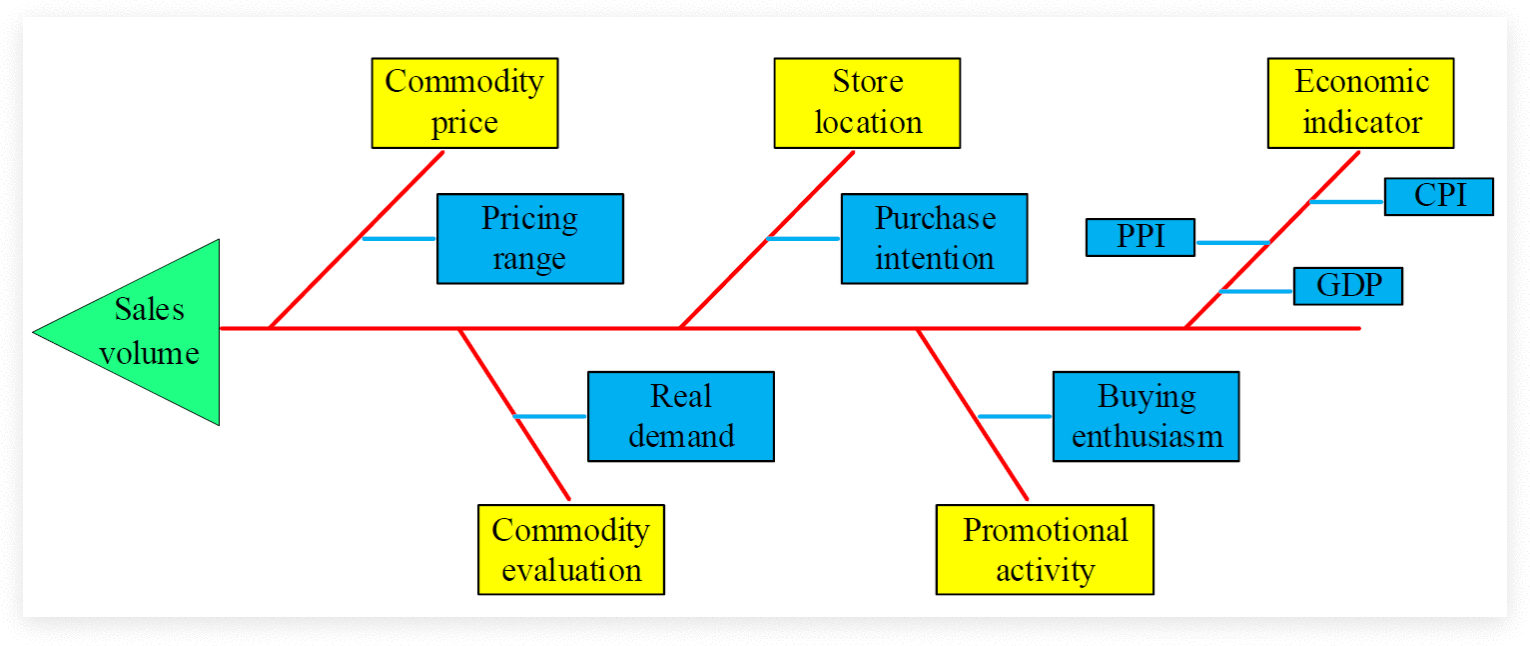
Factors affecting the sales volume of enterprise retail market.

Before predicting the retail prices of enterprises, it need to have a deep understanding of the influencing factors of the enterprise retail market. The accuracy of these factors and data will directly affect the effectiveness and results of the prediction model. In the retail process of enterprises, the current approach mainly adopts a combination of online and offline methods. The current sales factors affecting the retail market of enterprises mainly include product prices, product evaluations, store locations, promotional activities, and economic indicators [14,15]. The relevant diagram is shown in Fig 1.

Fig 1 shows the key factors influencing a company’s retail sales volume and their interrelationships. In this framework, the price of goods directly affects consumers’ purchasing intentions, among which the pricing range is one of the key factors. The location of a store influences consumers’ purchasing intentions by affecting their purchasing convenience, while economic indicators such as the Consumer Price Index (CPI), Producer Price Index (PPI), and Gross Domestic Product (GDP) affect consumers’ purchasing power and willingness at the macroeconomic level. In addition, product reviews reflect consumers’ satisfaction with the products, which not only affects real demand but may also grant the products higher pricing power. Promotional activities can boost sales in the short term by stimulating purchasing enthusiasm, and at the same time may enhance brand loyalty and market competitiveness. These factors interact with each other and jointly determine the sales volume and prices in the retail market. In enterprise retail, regression analysis is used to establish a relationship model between price and sales volume [16,17]. The relevant mathematical expression is shown in formula (1).


$$Q=\beta_{0}+\beta_{1}P+\varepsilon$$ (1)

In formula (1), Q represents sales volume, P represents price, $\beta_{0}$ and β represent regression coefficients, and ε represents price elasticity. Product evaluation is a reflection of consumer satisfaction with a product, usually presented in the form of ratings or reviews. Its mathematical expression is shown in formula (2).


$$Q=\beta_{0}+\beta_{1}R+\varepsilon$$ (2)

In formula (2), R represents the average rating of the product. The location of a store has a significant impact on retail sales, which can usually be quantified based on the location information of a company’s store, combined with factors such as population density and transportation convenience. Promotional activities can significantly increase sales, which can be quantified by the intensity and frequency of promotions. In addition, macroeconomic indicators also have an impact on retail market sales, reflecting the overall economic environment and thus affecting consumers’ purchasing power and willingness. The macroeconomic indicators involved in the study mainly include the CPI, which is an important index for measuring changes in consumer price levels, PPI, which measures changes in prices of goods and services in the production sector, and GDP, which measures a country’s economic situation. The mathematical expression of the sales model is shown in formula (3).


$$Q=\alpha_{0}+\alpha_{1}CPI+\alpha_{2}PPI+\alpha_{3}GDP+\varepsilon$$ (3)

**Fig 2:**
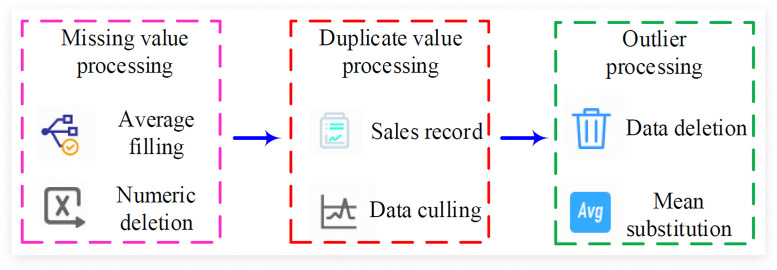
Data cleaning process.

In formula (3), $\alpha_{1}$, $\alpha_{2}$, and $\alpha_{3}$ represent regression coefficients. After clarifying the factors influencing the sales volume of enterprises, the actual operation data of a large chain retail enterprise in Sichuan Province, China, were studied and collected. The data covers the time span from January 2022 to December 2024, totaling 36 months of data. These data mainly include product codes, sales dates, store information, sales revenue, and activity intensity. After obtaining all the data, further data cleaning is carried out, including missing value processing, duplicate value processing, and outlier processing, as shown in Fig 2.

In Fig 2, common missing values in enterprise retail data may appear in fields such as product price, sales volume, promotional activities, etc [18]. Common missing values in the data may occur in fields such as commodity prices, sales volumes, and promotional activities. The research conducts corresponding processing based on the nature of the data and business requirements. If the percentage of missing values is less than 5%, and deletion will not significantly affect the analysis results, then records containing missing values can be deleted. Time series data such as promotional activity dates can be filled forward. For certain fields, such as promotional activity logos and product status fields, fixed value filling can be used [19]. Duplicate values may appear in retail data, especially during data merging or import processes. The handling of duplicate values can usually be identified by checking the uniqueness of the data to identify duplicate records. Alternatively, based on business requirements, it will choose to delete completely duplicate records and retain only one. The handling of outliers may be caused by data entry errors, measurement errors, or extreme situations. Outliers may be caused by data entry errors, measurement errors or extreme circumstances. The research adopts the Z-score method to identify outliers in the data. That is, after calculating the Z-score, a threshold is set to correct or delete the outliers, and its mathematical expression is shown in formula (4).


$$Z=\frac{x_{i}-\mu }{\sigma }$$ (4)

In formula (4), $x_{i}$ means the value of the data point, μ represents the mean, and σ represents the standard deviation of the dataset. After setting a threshold, the size of |Z| is determined. If it is greater than the threshold, the data point may be an outlier. After preprocessing the data, based on the continuous characteristics of enterprise data, the study first adopts a minimum maximum standardization method to scale the data to the range of [0,1]. Its mathematical expression is shown in formula (5).


$$X^{\sim }=\frac{x-\min(x)}{\max(x)-\min(x)}$$ (5)

In formula (5), min and max respectively represent the mini and max values in the dataset. Finally, the Spearman correlation coefficient is introduced to analyze the monotonic relationship between variables, and its mathematical expression is shown in formula (6).


$$r=\frac{\sum \left(X_{i}-X)(Y_{i}-Y\right)}{\sqrt{\sum {\left(X_{i}-X\right)}^{2}\sum {\left(Y_{i}-Y\right)}^{2}}}$$ (6)

In formula (6), r represents the correlation coefficient. Through correlation analysis, potential correlations between variables (such as price, sales volume, etc.) in enterprise retail data can be analyzed to further understand and obtain consistency in ranking.

### 2.2 Retail price prediction based on improved HPO-LSTM algorithm

**Fig 3:**
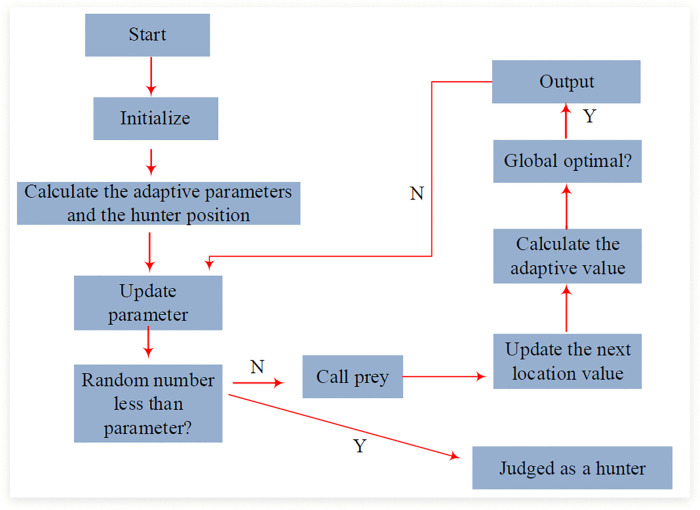
Flow chart of HPO algorithm.

After obtaining and processing enterprise retail data, the study introduces the Hunter-Prey Optimization (HPO) algorithm. It seeks the optimal solution by simulating the chase process between hunters and prey in nature, with the core idea of optimizing the search process based on the behavioral patterns of hunters and prey [20–22]. The process of HPO algorithm is shown in Fig 3.

In Fig 3, the initial positions of n individuals are randomly generated first, and the relevant mathematical expression is shown in formula (7).


$$x_{i}=l_{b}(u_{b}-l_{b})\cdot rand(0,1)$$ (7)

In formula (7), $l_{b}$ and $u_{b}$ mean the lower and upper bounds of the search space, respectively, and rand indicates the generated random number. After initializing the population, parameters such as population size, maximum iteration times, and adaptive parameter range are input, and the fitness of all individuals is calculated to find the initial optimal position. Subsequently, iterative optimization is carried out to update parameters C and Z. The mathematical expression of parameter C is shown in formula (8).


$$C=1-\frac{t}{MaxIt}\cdot (1-C_{\min})$$ (8)

In formula (8), t represents the iterative process, and MaxIt represents the max amount of iterations. The mathematical expression of parameter Z is shown in formula (9).


$$Z=Z_{\min}+rand(0,1)\cdot (Z_{\max}-Z_{\min})$$ (9)

After the parameter update is completed, for individual i, the role of their hunter or prey is determined by comparing random numbers and thresholds, as expressed mathematically in formula (10).


$$\left\{ \;\;\;\;\;\;\begin{array}{c} \begin{array}{cc} {}*20cx_{i}(t)+\alpha \cdot (P_{p}-x_{i}(t))+\beta \cdot (G_{best}-x_{i}(t)) & R≺\beta \end{array}\\ \begin{array}{cc} {}*20cx_{i}(t)+\gamma \cdot (R_{n}-x_{i}(t)) & otherwise \end{array} \end{array}\right.$$ (10)

In formula (10), R represents a random number within the range of [0,1], β represents a preset threshold, $P_{p}$ represents the current position of the prey, $G_{best}$ represents the global optimal position, and $R_{n}$ represents a randomly generated new position. After determining the individual’s role, their position in the search space is updated based on the role information. The mathematical expression of the hunter’s position information is shown in formula (11).


$$x_{hunter}(t+1)=x_{hunter}(t)+\alpha \cdot (P_{p}-x_{hunter}(t))+\beta \cdot (G_{best}-x_{hunter}(t))$$ (11)

The mathematical expression of the location information of the prey is shown in formula (12).


$$x_{prcy}(t+1)=x_{prcy}(t)+\gamma \cdot (R_{n}-x_{i}(t))$$ (12)

**Fig 4:**
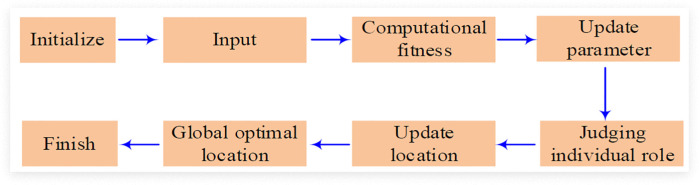
HPO-LSTM algorithm flow.

Finally, it will calculate the fitness of each individual and update the global optimal position. At the same time, it needs to check whether the termination condition is satisfied or the maximum number of iterations is reached, and output the optimal solution after the termination condition is satisfied. On the basis of HPO, the hyperparameters of LSTM recursive network are optimized grounded on this algorithm to raise the accuracy and efficiency of model prediction. After introducing the HPO algorithm, the LSTM model is initialized through the hyperparameter combination optimized by this algorithm. Meanwhile, the LSTM model is trained and its fitness is calculated on the validation set. Furthermore, after the optimization of the HPO algorithm, the global optimal position of the network model can be updated accordingly, and it can be checked whether the termination condition is met or the maximum number of iterations is reached. The specific process based on the HPO-LSTM algorithm is shown in Fig 4.

In Fig 4, in the improved HPO-LSTM, the HPO parameters are initialized first, setting the population size, the maximum number of iterations, and the threshold for hunters and prey; Initialize the LSTM parameters, that is, set the number of layers of the LSTM model, the number of neurons in each layer and the initial learning rate; Initialize the population, that is, randomly generate the hyperparameters of LSTM, and each group of hyperparameters represents an individual; Evaluate the fitness, that is, train the LSTM model using the current hyperparameter combination and calculate the fitness on the validation set; Update the HPO parameters, that is, update the positions of the hunter and the prey according to the fitness, and update the global optimal position; Check the termination condition, that is, if the maximum number of iterations is reached or the convergence condition is met, proceed to the next step; Otherwise, return to the “Evaluate Fitness” step. Optimize the LSTM model, that is, retrain the LSTM model using the best hyperparameters found by HPO; Finally, the prediction results are output, that is, the retail price prediction is carried out using the optimized LSTM model. Considering the existence of time series data in the retail price prediction process, this study further introduces attention mechanisms to optimize the HPO-LSTM model, to focus more on key information in the time series and enhance the accuracy and robustness of the prediction. The core of the attention mechanism lies in calculating the weight values for each time step, as expressed mathematically in formula (13).


$$w_{t}=\frac{\exp(e_{t})}{\sum i=1T\exp(e_{i})}$$ (13)

In formula (13), $w_{t}$ represents the attention weight for t time steps, $e_{t}$ represents the score for the t th time step, and T denotes the total length of the sequence. The score of the time step is usually calculated by the fully connected layer, and the relevant mathematical expression is shown in formula (14).


$$e_{t}=v^{T}\tanh(W_{h}h_{t}+W_{s}s_{t-1}+b)$$ (14)

In formula (14), $h_{t}$ stands for the hidden state of the t th time step, $s_{t-1}$ means the context vector of the previous time step, $W_{h}$, $W_{s}$, and v represent the weight matrices and vector, and b represents the bias term. After obtaining the attention weights, the model weights and sums the outputs of the LSTM based on the weight values to obtain the final output. The relevant mathematical expression is shown in formula (15).


$$h^{\sim }=\sum t=1Tw_{t}h_{t}$$ (15)

In formula (15), $h^{\sim }$ represents the weighted hidden state, and the model’s ability to capture key time steps is effectively improved after weighted summation. Specifically, in terms of input features, the input features are weighted through the attention mechanism. That is, after calculating the attention weights, the weighted features are input into the LSTM model, so that the model can pay more attention to the input features. In terms of the weighting of the hidden state, based on the attention weight as well, the weighted hidden state is taken as the output of the current time step. In terms of output weighting, through the attention weight, the weighted output is taken as the final prediction result to improve the accuracy of the model prediction. Finally, considering the unique gating mechanism of the LSTM model, the complexity of the model requires more time and computational resources during the training process. Therefore, the Q-learning algorithm is introduced to continuously iterate and update the value function to learn the optimal strategy. Firstly, a Q-Table is initialized and created to store all possible states, and based on the current Q-value, the action to be taken is decided. After selecting actions through a greedy strategy, the agent moves to a new state and receives rewards based on the results [23–25]. Finally, the Q-value of the state action pair is updated based on formula (16).


$$Q(s,a)=Q(s,a)+\eta [\nu +\kappa \cdot \max_{a^{\sim }}Q(s^{\sim },a^{\sim })-Q(s,a)]$$ (16)

**Fig 5:**
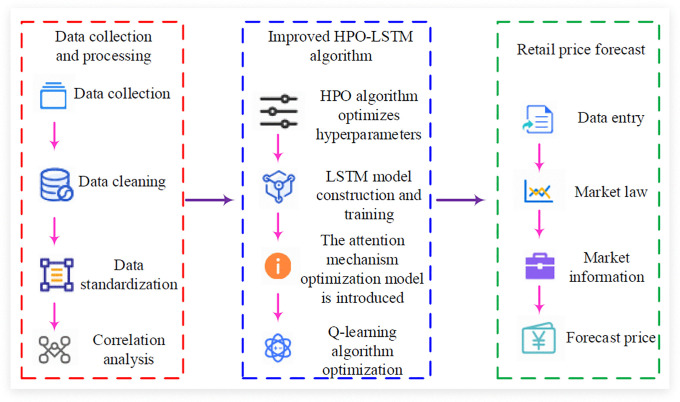
Business retail price forecasting process.

In formula (16), Q(s,a) represents the Q value corresponding to the current state and action, η represents the current learning rate, ν represents the reward for the action, κ represents the discount factor, and $\max_{a^{\sim }}Q(s^{\sim },a^{\sim })$ represents the max expected future reward for the next state. The Q-learning algorithm is introduced, and the optimal action can be selected by learning the optimal action value function (Q-function). Specifically, first initialize the Q-table, that is, create a Q-table to store the Q value of each state-action pair. Define the state and action Spaces, that is, the state is defined as the current parameter Settings and the current training error of the LSTM model during the training process, and the action is defined as the adjustment of the LSTM model parameters, that is, the adjustment of hyperparameters, etc. Secondly, during the training process, in each training step, select an action based on the current state. Finally, the learning rate of the LSTM model is dynamically adjusted through the Q-learning algorithm. If the optimal action in the current state is to increase the learning rate, then increase the learning rate in the next training step; Conversely, if the optimal action is to reduce the learning rate, then reduce the learning rate. Finally, the process of predicting retail prices for enterprises is shown in Fig 5.

In Fig 5, the prediction of enterprise retail prices mainly includes data collection and processing, the construction of an improved HPO-LSTM model, and the prediction of enterprise retail prices. In data collection, after collecting information such as product codes and sales figures, the data is cleaned and standardized to conduct correlation analysis on various indicators. In the improved HPO-LSTM model, hyperparameters are optimized using the HPO algorithm, and attention mechanism and Q-learning algorithm are introduced to optimize the model and training process. In the final retail price prediction stage, the processed data will be input into the optimized HPO-LSTM model.

Overall, the HPO algorithm, attention mechanism, and Q-learning algorithm were integrated into LSTM in the research, forming a novel prediction model. The HPO algorithm efficiently optimizes the hyperparameters of LSTM by simulating the chase process in nature, thereby enhancing the prediction accuracy of the model. The attention mechanism enables the model to focus on the key information in the time series, thereby enhancing the ability to capture long-term dependencies. The Q-learning algorithm optimizes the training process of the model by dynamically adjusting the learning rate and hyperparameters, and further improves the prediction performance. These three components complement each other and jointly solve the limitations of the standalone HPO-LSTM when dealing with complex time series data, improving the adaptability and generalization ability of the model. Compared with the standalone HPO-LSTM, the improved model performs better in terms of prediction accuracy and robustness, especially in the face of complex market environments and dynamic price fluctuations, providing a more efficient and accurate tool for enterprise retail price prediction.

## 3 Results

### 3.1 Correlation analysis of factors affecting market sales volume

Before model training, data preprocessing is a key step to ensure the performance of the model. The preprocessing process includes data cleaning (handling of missing values, duplicate values and outliers), data standardization (the minimal-maximum standardization method) and the method of feature selection. Among them, in terms of feature selection, based on the correlation analysis, the study selected the features that have a greater impact on retail prices. Before the experiment, multiple variable data including product prices, product evaluations, store locations, promotional activities, CPI, PPI, and GDP were collected. At the same time, correlation analysis was conducted on factors affecting sales in various markets based on the Spearman coefficient. The results are shown in Table 1.

**Table 1:** Correlation analysis of factors affecting market sales.

Index	Commodity price	Commodity evaluation	Store location	Promotional activity	CPI	PPI	GDP	Per customer	Conversion rate	Page view	Purchase increase rate	Market sales
Commodity price	1	−0.423*	−0.215	−0.654***	0.321*	0.412**	−0.123	−0.567***	−0.345*	−0.234	−0.854***	−0.854***
Commodity evaluation	−0.423*	1	0.345*	0.567***	−0.123	−0.098	0.234	0.456**	0.543***	0.321	0.721***	0.721***
Store location	−0.215	0.345*	1	0.456**	−0.098	−0.102	0.156	0.345*	0.234	0.123	0.632***	0.632***
Promotional activity	−0.654***	0.567***	0.456**	1	−0.234*	−0.156	0.345*	0.567***	0.456**	0.345*	0.887***	0.887***
CPI	0.321*	−0.123	−0.098	−0.234*	1	0.789***	−0.234*	−0.123	−0.098	−0.102	−0.213	−0.213*
PPI	0.412**	−0.098	−0.102	−0.156	0.789***	1	−0.123	−0.098	−0.102	−0.098	−0.156	−0.156
GDP	−0.123	0.234	0.156	0.345*	−0.234*	−0.123	1	0.234*	0.156	0.123	0.345*	0.345*
Per customer	−0.567***	0.456**	0.345*	0.567***	−0.123	−0.098	0.234*	1	0.567***	0.456**	0.567***	0.567***
Conversion rate	−0.345*	0.543***	0.234	0.456**	−0.098	−0.102	0.156	0.567***	1	0.345*	0.458***	0.458***
Page view	−0.234	0.321	0.123	0.345*	−0.102	−0.098	0.123	0.456**	0.345*	1	0.321	0.321
Purchase increase rate	−0.321*	0.456**	0.213	0.567***	−0.098	−0.102	0.156	0.543***	0.456**	0.567***	1	0.489***
Market sales	−0.854***	0.721***	0.632***	0.887***	−0.213*	−0.156	0.345*	0.567***	0.458***	0.321	0.489***	1

In Table 1, * indicates *p* < 0.05, ** indicates *p* less than 0.01, and *** indicates *p* < 0.001. In the results, Commodity price was significantly negatively correlated with Market sales (−0.854***), indicating that the higher the price, the lower the sales volume. This conforms to the basic laws in economics; Furthermore, it was significantly negatively correlated with Promotional activity (−0.654***) and with Per customer (−0.567***), indicating that promotional activities usually lower the price of goods. Meanwhile, the higher the price, the lower the average transaction value. Commodity evaluation was significantly positively correlated with Market sales (0.721***), indicating that the higher the consumers’ satisfaction with the commodity, the greater the possibility of purchase. Meanwhile, it was significantly positively correlated with Promotional activity (0.567***), indicating that promotional activities may enhance consumers’ evaluation of the goods. Promotional activity was significantly positively correlated with Market sales (0.887***), indicating that promotional activities can significantly increase sales volume. Store location was significantly positively correlated with Market sales (0.632***) and with Commodity evaluation (0.345*), indicating that store location has a significant impact on sales volume and may also affect consumers’ evaluations.

### 3.2 Performance evaluation based on improved HPO-LSTM algorithm

After determining the correlation of various influencing factors, further research was carried out to prove the effect of the improved HPO-LSTM algorithm. The experimental environment and algorithm parameter settings for the study are denoted in Table 2.

**Table 2:** Experimental environment and algorithm parameter setting.

Module	Model number	Argument	Value
CPU	Intel Core Ultra 9 285K	Batch processing	32
Internal memory	16GB	The initial learning rate of the LSTM model	0.01
Graphics card	NVIDIA GeForce GTX 3060	Number of neurons in the first hidden layer	100
Operating system	Windows 10 Professional	Number of neurons in the second hidden layer	50
Deep learning framework	PyTorch 1.6	Population size	40
Storage device	Samsung 980 PRO NVMe SSD 1TB	Max Iterations	100
/	/	Hunter-Prey Threshold	0.5
/	/	The number of layers of the LSTM model	2
/	/	Number of neurons per layer	100/50
/	/	The learning rate of the Q-learning algorithm	0.1
		Discount factor	0.9

In Table 2, an initial learning rate of 0.01 for the LSTM model is a commonly used option, which can enable the model to converge stably during the training process. A batch size of 32 can strike a balance between training efficiency and model performance. In the HPO algorithm, setting the population size to 40 could increase the diversity of the search space and improve the probability of finding the global optimal solution. Setting the hunter-prey threshold at 0.5 could enable the HPO algorithm to strike a balance between the behaviors of hunters and prey. Setting the discount factor of the Q-learning algorithm to 0.9 could strike a balance between short-term and long-term rewards. The number of layers and the number of neurons of the following control model BiLSTM are consistent with those of the LSTM model. The setting of 50 neurons per layer enabled the BiLSTM model to avoid overfitting when capturing forward and reverse time series information, while maintaining a high prediction accuracy. In addition, the learning rate and batch size are also consistent with the LSTM model. The Big Mart sales prediction dataset was selected, which includes sales data from large supermarkets, covering product features and sales volume for each product [26,27]. The ratio of the training set to the test set was set to 6:4. The relevant description of the dataset is shown in Table 3.

**Table 3:** Dataset description.

Dataset Feature	Description
Number of Samples	45000 samples
Number of Features	30 features
Feature Types	Numerical: 28:Categorical: 2
Target Variable	Retail Price
Time Period	January 2020 to December 2024
Missing Rate	5.20%
Data Source	A Large-scale Retail Chain Enterprise
Preprocessing Steps	Missing value treatment, outlier detection, normalization, standardization

**Fig 6:**
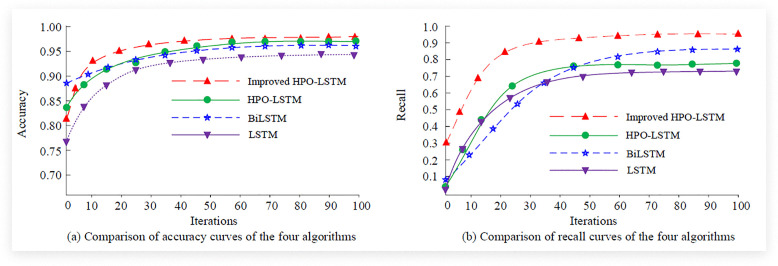
Comparison of accuracy and recall rate curves of four algorithms.

During the preprocessing of the model, missing value handling was carried out first. That is, numerical features and categorical features were filled with means and mode, respectively, and features with a missing rate lower than 5% were directly deleted. Secondly, the Z-score method was adopted to detect outliers, and they were corrected or deleted according to the business logic. Immediately after, normalize the data. Finally, based on the correlation analysis and feature importance assessment methods, the most influential features were selected, while the features with low correlation to the target variable were removed, thereby reducing the complexity of the model and the risk of overfitting. The training process of the model mainly included the training of the LSTM model optimized by the HPO algorithm. In the optimization of the HPO algorithm, the algorithm parameters and population were initialized first; Secondly, evaluate the combination of hyperparameters; Immediately afterwards, the positions of the hunter and the prey were updated according to the rules of the HPO algorithm, and the global optimal position was also updated. Finally, repeat the above steps until the maximum number of iterations is reached or the convergence condition is met. During the training process of the LSTM model, the LSTM model was initialized first using the optimal hyperparameters found by the HPO algorithm. Secondly, the LSTM model was trained on the training set and verified on the validation set to prevent overfitting. Immediately after that, the attention mechanism was introduced to enable the model to pay more attention to the key information in the time series; Finally, the Q-learning algorithm was used to dynamically adjust the learning rate and hyperparameters to optimize the training process. The study simultaneously selected traditional HPO-LSTM, LSTM, and BiLSTM algorithms for comparative experiments. Among them, the BiLSTM had 2 layers, the number of neurons was 100 and 50 respectively, the learning rate was 0.01, the batch size was 32, and the Adam optimizer was also adopted. Firstly, the accuracy and recall curves of the four algorithms are denoted in Fig 6.

**Fig 7:**
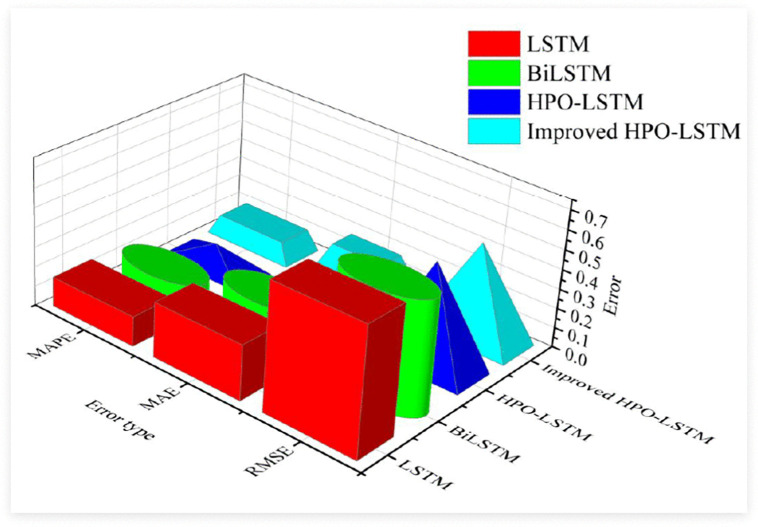
Comparison of error values of the four algorithms.

Fig 6 (a) compares the accuracy curves of four algorithms. The outcomes showed that the improved HPO-LSTM algorithm used in the study had the highest accuracy after convergence, approaching 0.972, followed by the traditional HPO-LSTM model, BiLSTM model, and traditional LSTM model. Fig 6 (b) shows a comparison of recall curves for four algorithms. The findings showed that the improved HPO-LSTM algorithm used in the study had the highest recall rate, approaching 0.921. Meanwhile, the initial recall rate of this algorithm was 0.301, which was significantly higher than other algorithms. The study further compared the three error values of the four algorithms, and the findings are denoted in Fig 7.

Fig 7 compares the error values of four algorithms. The findings denoted that the three error values of the improved HPO-LSTM algorithm proposed in the study were the smallest, with RMSE, MAE, and MAPE of 0.48, 0.20, and 0.105, respectively. This indicated that the algorithm had high accuracy and small error in predicting enterprise retail prices. The traditional HPO-LSTM algorithm and BiLSTM algorithm had the second highest error values, while the traditional LSTM algorithm had the highest error value. The study further compared the computational efficiency and complexity of the four algorithms, and the results are shown in Table 4.

**Table 4:** Comparison of computational efficiency and computational complexity.

Training Set Size	Improved HPO-LSTM	Traditional HPO-LSTM	LSTM	BiLSTM
Runtime (seconds)	/	/	/	/
10000 samples	120.5	135.2	90.3	105.4
20000 samples	230.7	250.9	180.6	210.8
30000 samples	340.9	375.4	270.9	315.2
40000 samples	451.2	500.1	360.4	420.3
50000 samples	561.5	625.3	450	525.4
Memory Usage (MB)	/	/	/	/
10000 samples	1251	1351	1036	1148
20000 samples	2354	2541	1836	2147
30000 samples	3400	3700	2700	3158
40000 samples	4517	5027	3647	4203
50000 samples	5614	6235	4531	5247

**Fig 8:**
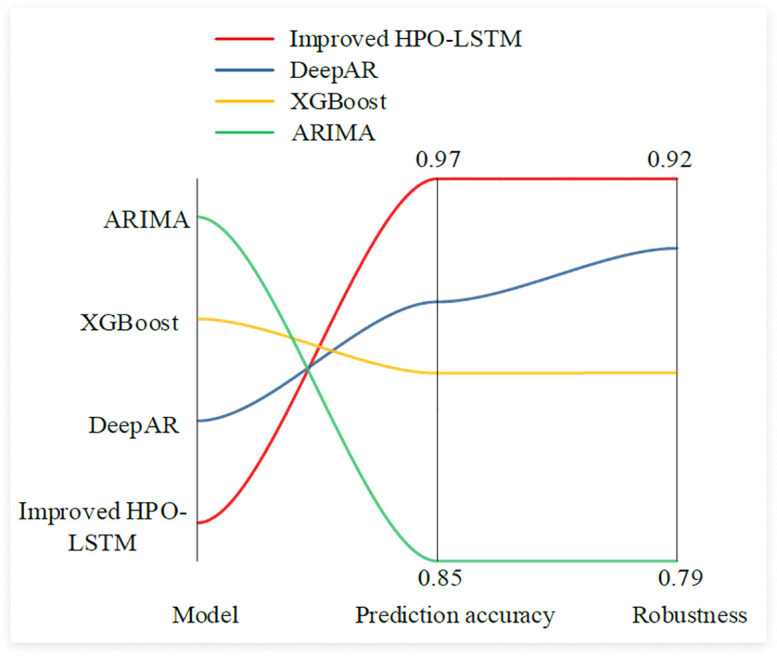
Comparison of the prediction accuracy and robustness of the four models.

In Table 4, although the improved HPO-LSTM algorithm had a slightly higher running time than the traditional LSTM and BiLSTM, its running time was significantly shorter compared with the traditional HPO-LSTM. For example, under the training set of 50,000 samples, the running time of the improved HPO-LSTM was 561.5 seconds, while that of the traditional HPO-LSTM was 625.3 seconds. The running time of the improved algorithm decreased by approximately 10.2%, indicating that the improved algorithm is more efficient in the optimization process and can find the appropriate hyperparameters more quickly, thereby reducing the overall training time. In terms of memory usage, although the improved HPO-LSTM algorithm had higher memory usage than the traditional LSTM and BiLSTM, compared with the traditional HPO-LSTM, the memory usage was significantly reduced. For example, under a training set of 50,000 samples, the memory usage of the improved HPO-LSTM was 5,614 MB, while that of the traditional HPO-LSTM was 6,235 MB. The memory usage of the improved algorithm was reduced by approximately 9.9%. It indicates that the improved algorithm effectively controls the memory occupation during the optimization process and reduces unnecessary memory consumption. At the end of the study, the autoregressive integral moving average model (ARIMA), XGBoost, and DeepAR model were introduced for control experiments. Among them, DeepAR had 2 layers, with 50 neurons in each layer, a learning rate of 0.01, a batch size of 32, and also used the Adam optimizer. The comparison of the prediction accuracy and robustness of the four models is shown in Fig 8.

**Fig 9:**
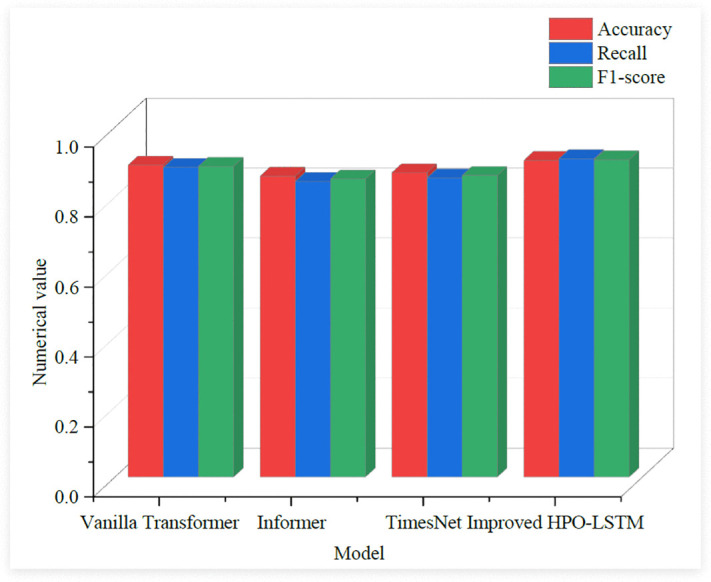
Comparison of model performance results.

In Fig 8, the prediction accuracy of the improved HPO-LSTM was 97.20%, which performs the best among all models. This indicates that the improved HPO-LSTM model can be very close to the actual value when predicting retail prices and has high prediction accuracy. Meanwhile, the robustness index was 92.10%, which also performs the best among all models. This indicates that the stability of this model is very high under different datasets and different conditions, and it can adapt to various complex situations. The prediction accuracy and robustness indicators of the DeepAR model were second only to those of the improved HPO-LSTM model, with values of 93.40% and 89.70% respectively. The ARIMA model performed the worst among all models. Its prediction accuracy and robustness were 85.40% and 78.90% respectively, indicating that its prediction accuracy is relatively low in the case of a complex market environment and the interaction of multiple factors. Meanwhile, it had poor stability and was easily affected by data noise and complex situations. The study further introduced three relatively advanced time series models for comparative experiments, namely Vanilla Transformer, Informer and TimesNet. The performance results comparison is shown in Fig 9.

In Fig 9, the improved HPO-LSTM model outperformed the three variants of the Transformer model in the three key performance metrics of accuracy, recall and F1-score. Its accuracy was 0.905, the recall was 0.910 and the F1-score was0.907. In contrast, although the Vanilla Transformer model performed well in time series prediction, its performance was slightly lower than that of the improved HPO-LSTM model. This might be because when the Vanilla Transformer deals with time series data with complex dependencies. Its self-attention mechanism may not be able to fully capture all the key information. Although the Informer and TimesNet models have been optimized for long sequence prediction, they are still slightly inferior to the improved HPO-LSTM model in terms of accuracy and recall. This might be because they may have certain limitations when dealing with specific types of data. At the end of the study, the influence of each component on the model performance was analyzed through ablation experiments. The results of the ablation experiment are shown in Table 5.

**Table 5:** Results of the ablation experiment.

Model Configuration	Accuracy	Recall	F1 Score
Baseline LSTM	0.845	0.83	0.837
+ HPO Optimization	0.87	0.86	0.865
+ Attention Mechanism	0.875	0.865	0.87
+ Q-learning	0.88	0.87	0.875
Full Model (HPO + Attention + Q-learning)	0.905	0.91	0.907

In Table 5, as each component in the model was gradually added, the prediction performance indicators all showed a gradually improving trend. The performance of the specific basic LSTM model in terms of accuracy, recall rate and F1-score was 0.845, 0.830 and 0.837 respectively. After the introduction of HPO optimization, the performance indicators were respectively improved to 0.870, 0.860 and 0.865, indicating that HPO optimization has played a significant role in enhancing the model’s performance. After further introducing the attention mechanism, the performance indicators improved again, indicating that the attention mechanism helps the model better capture key information, thereby enhancing the accuracy of predictions. Finally, after incorporating the Q-learning algorithm, the performance of the model was further enhanced. When all components were fully integrated, the predictive performance of the model reached its optimum, with accuracy, recall rate and F1-score as high as 0.905, 0.910 and 0.907, respectively.

### 3.3 Verification of prediction capability and analysis of practical business application effect of improved HPO-LSTM algorithm

**Fig 10:**
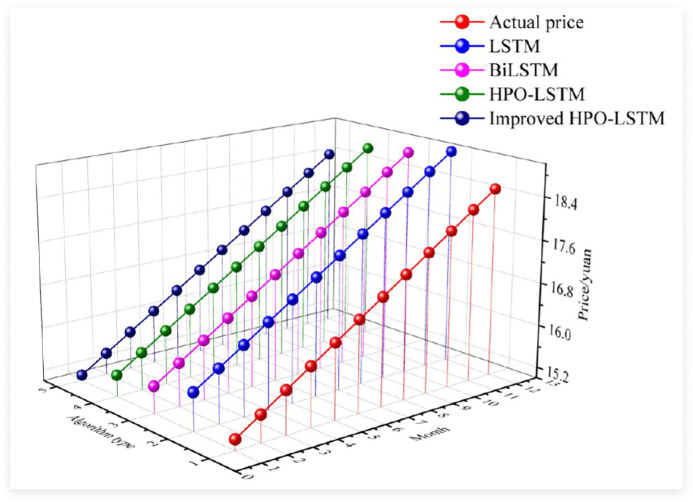
The difference between the four algorithms and the actual price.

After verifying the performance of the four algorithms, the study further focused on the price fluctuations of a certain product from January to December 2024. By comparing the differences between the four algorithms and the actual prices, the retail price prediction ability of the algorithms was validated. The results are shown in Fig 10.

**Fig 11:**
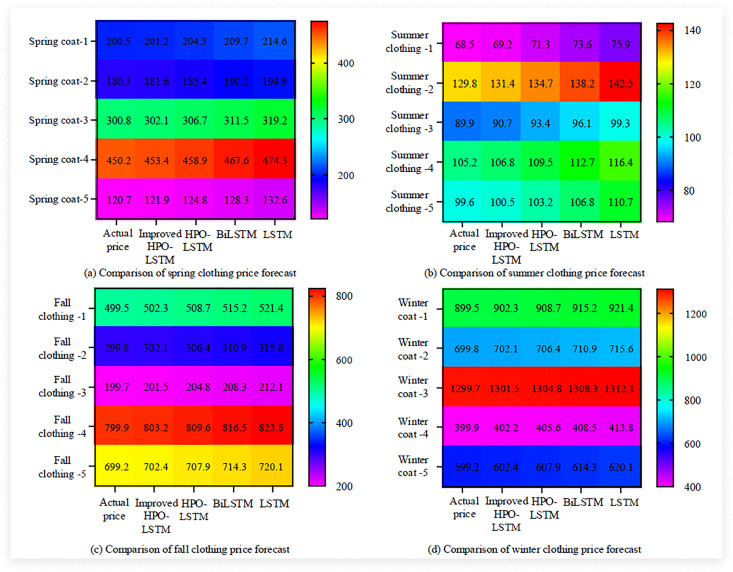
Comparison of price forecast results of four kinds of seasonal products.

Fig 10 shows the comparison between four algorithms and the actual price of a certain product. The results indicated that the price of the product was generally between 15–18 yuan. The improved HPO-LSTM algorithm had the closest prediction results to the actual price with the smallest error. For example, in January, the actual price was 15.2 yuan, and the improved HPO-LSTM predicted a value of 15.1 yuan. The traditional HPO-LSTM prediction results were slightly higher than the actual price, but the overall error was still smaller than BiLSTM and traditional LSTM. The predicted values of BiLSTM were generally higher than the actual prices, with slightly larger errors than the improved HPO-LSTM. The difference between the latest LSTM prediction value and the actual price was significant, with the highest error. Overall, the improved HPO-LSTM algorithm performs the best in retail price prediction, indicating significant advantages in hyperparameter optimization and time series feature capture. The study further compared a certain seasonal product of the enterprise in the four seasons of spring, summer, autumn, and winter, and the price prediction effect based on the four products is shown in Fig 11.

**Fig 12:**
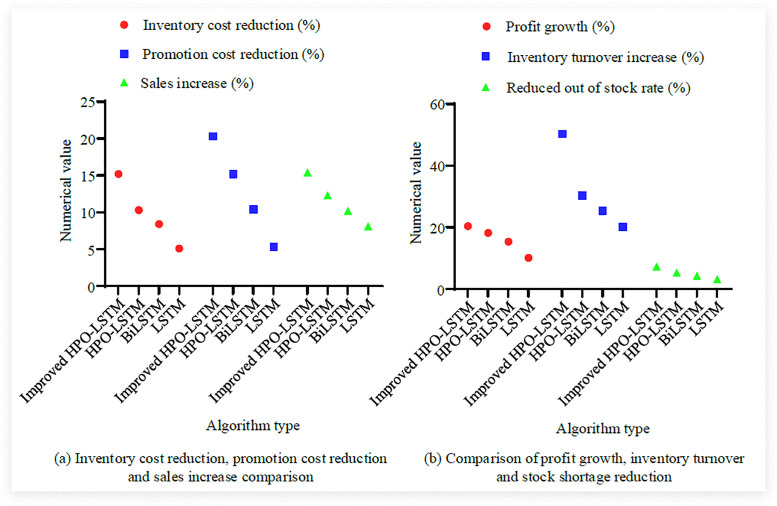
Comparison of actual service data based on four algorithms.

Figs 11 (a), 11 (b), 11 (c), and 11 (d) respectively show the comparison of retail price prediction algorithms for four seasons: spring, summer, autumn, and winter. The findings denoted that the improved HPO-LSTM algorithm exhibited the highest accuracy in predicting all seasons, with the smallest error between predicted prices and actual prices. On spring coat 1, the actual price was 200.5 yuan, and the improved HPO-LSTM algorithm predicted 201.2 yuan, with an error of only 0.7 yuan. Meanwhile, the improved HPO-LSTM algorithm had a smaller error range. For example, on summer clothing 1, the error was only 0.47 yuan, while the error of the LSTM algorithm reached7.4 yuan; On the winter coat 3, the error of the improved HPO-LSTM algorithm was only 1.8 yuan. Finally, the improved HPO-LSTM showed high stability in different seasons, price ranges, and product types, with errors of less than 1 yuan in low-priced products in spring and autumn. In the high priced winter products, such as the improved HPO-LSTM on Winter Coat 3, the error was only 1.8 yuan, significantly lower than other algorithms. Finally, the actual business data of four algorithms were compared and the results are shown in Fig 12.

Fig 12 (a) shows a comparison of inventory cost reduction, promotion cost reduction, and sales revenue increase. The findings denoted that the improved HPO-LSTM significantly reduced inventory backlog and inventory costs by 15.2% by accurately predicting demand. At the same time, by reducing unnecessary promotional frequency, promotional costs were reduced by 20.3%; In addition, the improvement of HPO-LSTM significantly increased sales (15.4%) by optimizing product pricing and promotion strategies, while also increasing average order value and sales volume. Fig 12 (b) shows a comparison of profit growth, inventory turnover improvement, and shortage rate reduction. The results showed that improved HPO-LSTM directly promoted enterprise profit growth (20.4%), while the profit growth of LSTM was only 10.1%. Simultaneously, accurately predicting demand using improved HPO-LSTM reduced inventory backlog and increased inventory turnover by 50.3%, significantly outperforming other algorithms; Finally, by making more accurate price predictions, the shortage phenomenon was reduced, and the shortage rate decreased by 7.2%.

## 4 Discussion and conclusion

To improve the accuracy and efficiency of enterprise retail price prediction, the HPO algorithm combined with LSTM algorithm was studied, and attention mechanism and Q-learning algorithm were introduced to optimize the HPO-LSTM algorithm. The optimized algorithm was applied to the prediction of enterprise retail prices. In the research results, there was a significant negative correlation (−0.854) between product prices and market sales, indicating that price is a key factor affecting sales. In addition, product evaluation was positively correlated with market sales (0.721), indicating that consumer satisfaction with the product directly affects purchasing decisions. In terms of performance evaluation of the algorithm, the improved HPO-LSTM had the highest accuracy and recall, with converged values of 0.972 and 0.921, respectively. This is similar to the research results in reference [28], which optimized the prediction model of the nonlinear autoregressive neural network to make the model more accurate in predicting products such as crude oil. Unlike the research, this study not only optimized the parameters but also optimized the network structure of the model. In terms of predicting retail prices for enterprises, the improved HPO-LSTM algorithm had the best predictive ability, with an error of only 0.7 yuan in spring coat 1. In winter coat 3, the error was 1.8 yuan. Finally, in practical business applications, the improved HPO-LSTM algorithm could significantly reduce inventory costs (15.2%) and promotion costs (20.3%), while increasing sales revenue (15.4%). This is basically consistent with the results of Harshith N et al., who predicted the annual price of a certain product in 2022 through a stacked LSTM model. The results show that after using this prediction model, the cost has been reduced to a certain extent. Unlike research, reference [29] focused on the agricultural field in terms of research background and objectives, especially the price prediction of cumin. At the same time, this study used time-series data from 2002 to 2021, with a larger time span and data volume; However, reference [26] did not show significant growth in sales, which may be due to the failure of the company’s business strategy to optimize and adjust according to the predicted results, resulting in a less significant effect on the increase in sales.

The above research results indicate that the improved HPO-LSTM algorithm performs well in prediction accuracy and robustness, and can maintain high prediction accuracy in different seasons, price ranges, and product types. At the same time, it can bring significant economic benefits to enterprises, such as reducing inventory costs, promotion costs, and increasing sales and profits. However, despite achieving good results in the research, there are still certain shortcomings. There is still room for improvement in terms of data volume and time span, and the generalization ability of the model has not been fully validated in a wider market environment. Future research will expand the dataset size, further optimize the model structure to raise its adaptability and generalization ability in different market environments, and explore more dynamic relationships between influencing factors and retail prices, providing more comprehensive support for accurate pricing and market strategies of enterprises.
